# Combined Fixed and Dynamic Left Ventricular Outflow Tract Obstruction in Hypertrophic Cardiomyopathy Due to a Coexisting Subaortic Membrane: A Case Report

**DOI:** 10.3390/jcm15083115

**Published:** 2026-04-19

**Authors:** Katherine Zambrano-Cevallos, Silvia Zurita-Fuentes, Liliana Cardenas, Luis Miguel Guerrero, Alejandra García, Juan Jaramillo-Merino, Sofía Gavilánez-Zambrano, Marlon Rojas-Cadena, Juan S. Izquierdo-Condoy

**Affiliations:** 1 Department of Cardiology, Hospital de Especialidades Eugenio Espejo, Quito 170403, Ecuador; 2 One Health Research Group, Universidad de Las Américas, Quito 170124, Ecuador

**Keywords:** hypertrophic cardiomyopathy, subaortic membrane, subaortic stenosis, systolic anterior motion (SAM), mitral regurgitation, left ventricular outflow tract obstruction (LVOTO)

## Abstract

**Introduction:** Hypertrophic cardiomyopathy (HCM) is a common myocardial disease worldwide and is associated with heart failure symptoms and sudden cardiac death. In a subset of patients, it may produce dynamic left ventricular outflow tract obstruction (LVOTO) and systolic anterior motion (SAM)-related mitral valve dysfunction through drag forces and altered mitral–septal geometry. In contrast, subaortic stenosis caused by a subaortic membrane is an uncommon congenital lesion that may lead to fixed subvalvular LVOTO in adulthood. The coexistence of these entities is rare and can substantially complicate diagnosis and management. **Case presentation:** A 51-year-old woman with HCM, paroxysmal atrial fibrillation, and heart failure presented with acute decompensation and cardiogenic shock. After initial hemodynamic stabilization and cardioversion for atrial fibrillation with rapid ventricular response, multimodality imaging with transthoracic and transesophageal echocardiography, coronary computed tomography angiography, and cardiac magnetic resonance demonstrated dual LVOTO, with a dynamic component related to HCM/SAM physiology and a fixed component caused by an elongated subaortic membrane, accompanied by severe SAM-related mitral regurgitation. Echocardiography showed a resting peak LVOT gradient of 49 mmHg, increasing to 85 mmHg with the Valsalva maneuver. After exclusion of obstructive coronary artery disease and evaluation for selected phenocopies, the patient underwent septal myectomy, subaortic membrane resection, and adjunctive mitral valve plication. Early postoperative echocardiography showed reduction in the maximum provoked LVOT gradient to 38 mmHg and improvement of mitral regurgitation from severe to mild. At 3-month follow-up, she remained in sinus rhythm, improved to New York Heart Association functional class II, and had no documented readmissions for heart failure. **Conclusions:** Combined fixed and dynamic LVOTO due to concomitant subaortic membrane and HCM is exceedingly rare. Accurate diagnosis requires a high index of suspicion and a multimodality imaging strategy to define the obstructive mechanisms and support mechanism-based surgical management and avoid incomplete treatment when a coexisting fixed lesion is present.

## 1. Introduction

Hypertrophic cardiomyopathy (HCM) is a common disease reported in populations globally. It is often inherited with complex phenotypic and genetic expression and a variable natural history, affecting both genders, although women are diagnosed less commonly than men. HCM is defined as the presence of increased LV wall thickness (with or without RV hypertrophy) or mass that is not solely explained by another cardiac, metabolic, or systemic (syndromic) disease capable of producing a similar magnitude of hypertrophy [1].

Subaortic stenosis is a rare form of left ventricular outflow tract obstruction (LVOTO), accounting for approximately 6.5% of adult congenital heart disease and representing the second most common cause of aortic stenosis, responsible for nearly 14% of left ventricular outflow tract obstructions [2].

These two conditions may manifest with symptoms including presyncope, exertional dyspnea, and fatigue and, with disease progression, chest pain or syncope occurring during physical exertion or at rest. In advanced stages, subaortic stenosis may ultimately lead to congestive heart failure [3,4].

The coexistence of these two conditions is extremely rare, and the presence of LVOTO resulting from both entities is particularly uncommon, with only a limited number of cases previously reported in the literature [5,6,7,8].

The diagnosis may be challenging when two distinct pathological entities affecting the left ventricular outflow tract coexist and produce combined obstruction, as the resulting clinical manifestations are often similar. Even in experienced centers, this underscores the critical role of multimodality imaging in the accurate diagnosis of complex and overlapping disease processes [6,9].

Here, we report an extremely rare case of combined fixed and dynamic LVOTO and highlight the diagnostic and therapeutic implications of a multimodality imaging approach. We describe a patient with hypertrophic cardiomyopathy and dynamic LVOTO coexisting with a subaortic membrane causing fixed subvalvular obstruction, complicated by severe systolic anterior motion-related mitral regurgitation, who presented with acute heart failure and cardiogenic shock attributable to this dual obstructive physiology.

## 2. Case Presentation

A 51-year-old woman with a history of hypertrophic cardiomyopathy with subvalvular outflow tract obstruction and mitral regurgitation presented with cardiogenic shock. She reported a history of paroxysmal atrial fibrillation managed with rivaroxaban for anticoagulation and bisoprolol (1.25 mg) for heart rate control. Additionally, she had heart failure with preserved ejection fraction (HFpEF), characterized by worsening functional capacity, progressing from New York Heart Association (NYHA) Class II to Class III. Her heart failure regimen included furosemide (40 mg). She denied any family history of cardiac or other relevant systemic diseases. The patient reported experiencing symptomatic heart disease for approximately one year, including intermittent precordial chest pain, presyncope, dizziness, and palpitations. Due to symptom progression and complex valvular disease, she was awaiting cardiac surgical intervention.

**Figure 1:**
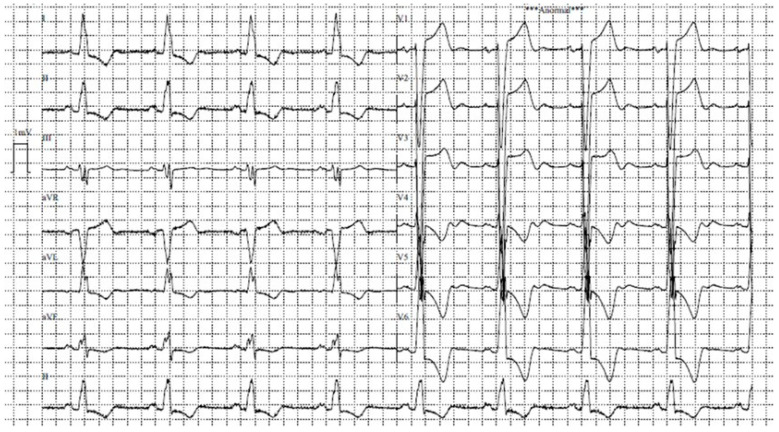
Twelve-lead electrocardiogram after cardioversion. Twelve-lead ECG showing sinus rhythm with complete left bundle branch block, QRS fragmentation in leads III, aVF, and aVL, and secondary repolarization abnormalities.

The patient was re-presented to the emergency department due to cardiogenic shock, necessitating the initiation of vasopressor and inotropic support with norepinephrine and dobutamine. Physical examination was consistent with significant cardiac compromise and congestion. Heart sounds were irregular due to atrial fibrillation with rapid ventricular response. A Grade IV/VI systolic ejection murmur radiating to the suprasternal notch was noted, suggestive of significant LVOTO and/or aortic outflow turbulence. A Grade II/VI holosystolic murmur radiating to the axilla was consistent with mitral regurgitation. Decreased breath sounds at both lung bases, bilateral crackles, and lower extremity edema (+++/++++) indicated severe pulmonary and systemic congestion. Baseline laboratory results are summarized in Table 1. The initial electrocardiogram confirmed atrial fibrillation with rapid ventricular response, aberrant conduction, and left bundle branch block. Given the hemodynamic instability, electrical cardioversion was performed, followed by attempted pharmacological cardioversion using amiodarone (a class III antiarrhythmic agent). This approach successfully restored sinus rhythm, as shown in Figure 1. The patient achieved hemodynamic stability and was maintained on minimal doses of vasoactive drugs. These agents were weaned and discontinued within 48 h, with the patient maintaining adequate end-organ perfusion.

**Figure 2:**
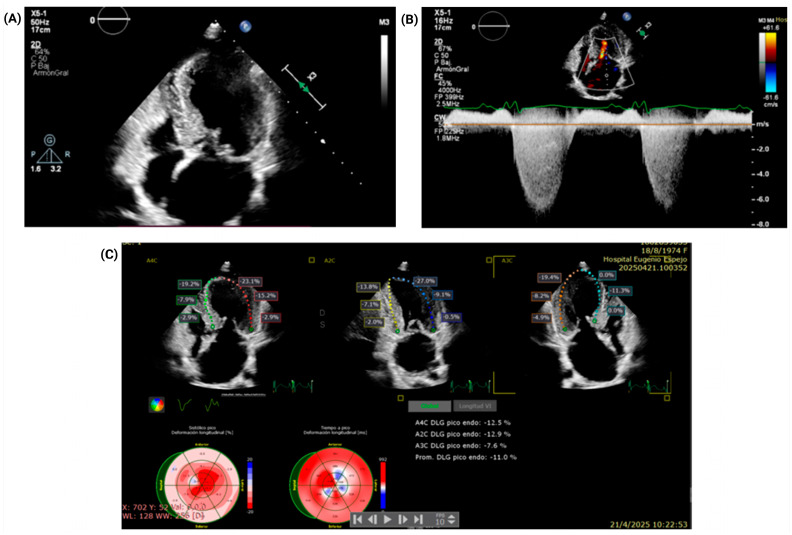
Transthoracic echocardiography: structural, hemodynamic, and deformation findings. (**A**) Two-dimensional transthoracic echocardiography demonstrating asymmetric septal hypertrophy, predominantly involving the anteroseptal and inferoseptal walls (basal to mid-ventricular segments), with a maximal end-diastolic wall thickness of 18 mm. (**B**) Doppler assessment of mitral inflow showing estimated peak and mean diastolic left atrial–left ventricular gradients of 19 mmHg and 6 mmHg, respectively; severe mitral regurgitation was present, with a peak systolic left ventricular–left atrial regurgitant velocity of 6.4 m/s, consistent with systolic anterior motion-related mitral regurgitation. (**C**) Speckle-tracking echocardiography demonstrating markedly reduced global longitudinal strain (GLS), with a reported GLS value of −11%.

Following hemodynamic stabilization, transthoracic echocardiography (TTE) was performed to further evaluate the underlying structural pathology. The TTE demonstrated asymmetric septal hypertrophy with predominance in anteroseptal and inferoseptal walls, with a maximum wall thickness of 18 mm, raising suspicion for a dual LVOTO mechanism (fixed and dynamic) due to the presence of a subaortic membrane and hypertrophic cardiomyopathy (Figure 2). Transthoracic echocardiography demonstrated a resting peak LVOT gradient of 49 mmHg (peak velocity 3.49 m/s), which increased to 85 mmHg with the Valsalva maneuver (peak velocity 4.61 m/s), supporting a dynamic component of obstruction. However, the relative hemodynamic contribution of the fixed and dynamic components could not be fully quantified retrospectively, because separate serial Doppler envelopes, post-extrasystolic gradients, and detailed continuous-wave Doppler contour analysis were not available. The aortic valve was trileaflet, without significant stenosis, with a valve area of 1.59 cm^2^ and an indexed valve area of 1.15 cm^2^/m^2^. Other key findings included severe mitral regurgitation, with an eccentric jet reaching the roof of the left atrium on color Doppler and a peak regurgitant velocity of 6.4 m/s. The regurgitation was interpreted as predominantly SAM-related. The mitral annulus was dilated, measuring 35 × 36 mm. The left atrium was severely dilated, with a linear dimension of 62 mm and a volume index of 89 mL/m^2^. Biventricular systolic function was preserved, with a left ventricular ejection fraction of 65%, a TAPSE of 22 mm, and a fractional area change of 45%. However, a more sensitive measure of contractility showed a markedly reduced global longitudinal strain (GLS) of −11%.

**Figure 3:**
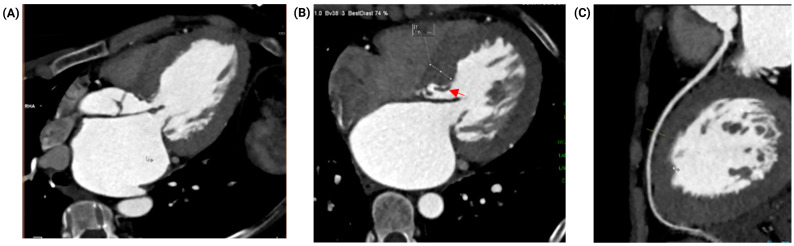
Coronary computed tomography angiography and anatomic definition of the LVOT. (**A**) CT findings consistent with an elongated subaortic membrane producing fixed subvalvular LVOT obstruction. (**B**) Marked left atrial and left ventricular dilatation with interventricular septal hypertrophy. (**C**) Coronary arteries without evidence of obstructive coronary artery disease. Red arrow indicates the elongated subaortic membrane.

Coronary computed tomography angiography (CCTA) revealed no evidence of obstructive coronary artery disease (CAD). The study showed a coronary artery calcium (CAC) score of zero (0) and no atherosclerotic plaque, resulting in a CAD-RADS score of 0 for all three coronary arteries (Figure 3). Furthermore, the CT scan provided crucial structural details, demonstrating an elongated subaortic membrane producing fixed subvalvular LVOT obstruction, severe bi-atrial and left ventricular dilation, and no evidence of thrombus within the left atrial appendage.

**Figure 4:**
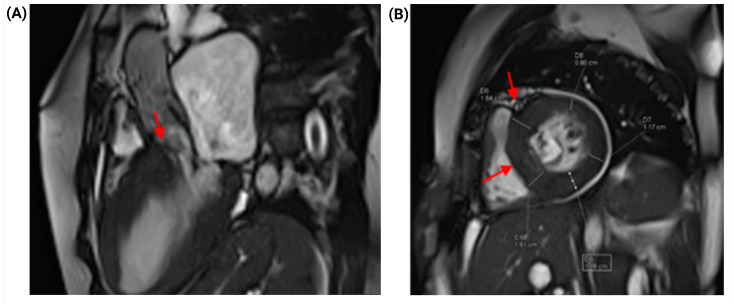
Cardiac magnetic resonance imaging: structural characterization and myocardial fibrosis. (**A**) Cardiac magnetic resonance image demonstrating an elongated subaortic membrane (red arrow) consistent with fixed subvalvular LVOT obstruction at rest. (**B**) Short-axis cardiac magnetic resonance image showing asymmetric hypertrophic cardiomyopathy with predominant septal hypertrophy (maximum septal thickness 16.4 mm; red arrows) and myocardial tissue characterization demon-strating late gadolinium enhancement, quantified as 13% myocardial fibrosis.

Cardiac magnetic resonance imaging (CMR) provided additional structural and tissue characterization that supported the diagnosis. A filamentous subvalvular structure was identified 9 mm from the aortic annular plane, measuring 17 mm in length, consistent with a subaortic membrane capable of producing significant fixed subvalvular obstruction. Systolic turbulence was observed within the left ventricular outflow tract (LVOT), with qualitative evidence of dynamic LVOT obstruction at rest. Asymmetric myocardial hypertrophy with anteroseptal and inferoseptal predominance was confirmed, with a maximum diastolic wall thickness of 16 mm, consistent with hypertrophic cardiomyopathy (HCM). Quantification of myocardial fibrosis by late gadolinium enhancement (LGE) demonstrated an LGE burden of 13%, indicating extensive replacement fibrosis (Figure 4).

**Figure 5:**
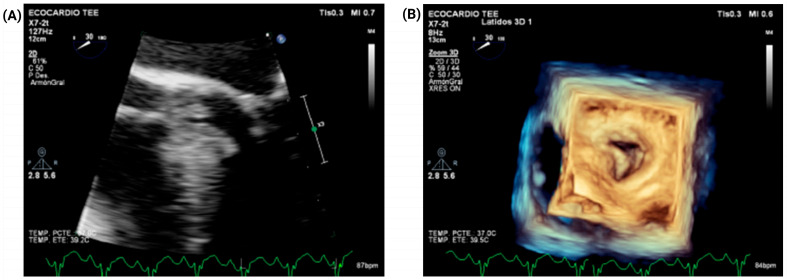
Transesophageal echocardiography: dual-mechanism LVOT obstruction. (**A**) Transesophageal echocardiography showing an elongated subaortic membrane contributing to fixed subvalvular LVOT obstruction. (**B**) Representative imaging illustrating dual-mechanism LVOT obstruction, with a fixed component due to the subaortic membrane and a dynamic component related to HCM physiology, including systolic anterior motion.

Transesophageal echocardiography (TEE) further corroborated the diagnosis, demonstrating asymmetric septal hypertrophy and LVOTO. TEE also confirmed severe mitral regurgitation, interpreted as predominantly SAM-related. No evidence of rheumatic valvular disease or other clear intrinsic leaflet abnormality was identified. The study also demonstrated an elongated subaortic membrane contributing to fixed subvalvular LVOT obstruction (Figure 5). In conjunction with transthoracic echocardiography, these findings also showed annular dilatation, suggesting an additional geometric contribution to MR severity beyond SAM alone. On this basis, the Heart Team elected to perform adjunctive mitral valve plication to optimize leaflet coaptation and reduce residual postoperative MR. Complete quantitative leaflet geometry measurements and detailed operative valve analysis were not available in the archived record.

Due to the atypical obstructive physiology and the coexistence of a subaortic membrane, the Heart Team elected to proceed with genetic testing. Given the atypical phenotype and complex obstructive physiology, etiologic evaluation included sarcomeric gene testing and assessment for selected phenocopies. No pathogenic sarcomeric variants were identified, and ancillary evaluation for Fabry disease and transthyretin amyloidosis was unrevealing.

Given persistent symptoms, recurrent decompensation, and clinically significant obstruction attributable to both septal hypertrophy and the subaortic membrane, the patient underwent surgical treatment after multidisciplinary Heart Team discussion. A combined surgical strategy was selected to address each lesion according to its mechanism: subaortic membrane resection for the fixed component, septal myectomy for the dynamic HCM-related component, and adjunctive mitral valve plication because marked annular dilatation (35 × 36 mm) was considered to contribute to MR severity beyond SAM alone. The patient tolerated the procedure well, with early postoperative symptomatic improvement.

**Figure 6:**
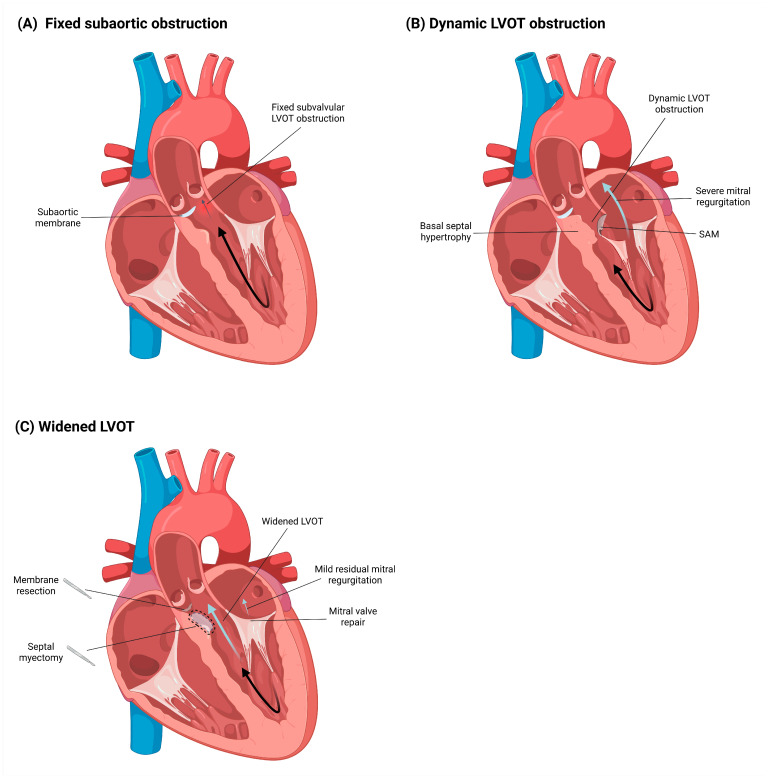
Schematic representation of combined fixed and dynamic left ventricular outflow tract obstruction and mechanism-based surgical correction. (**A**) Fixed subaortic obstruction caused by a subaortic membrane located below the aortic valve. (**B**) Dynamic LVOT obstruction caused by basal septal hypertrophy and systolic anterior motion (SAM) of the anterior mitral leaflet, resulting in dynamic narrowing of the LVOT and severe mitral regurgitation. (**C**) Mechanism-based surgical correction with subaortic membrane resection for the fixed component, septal myectomy for the dynamic HCM-related component, and adjunctive mitral valve plication, resulting in widening of the LVOT and mild residual mitral regurgitation. The arrows in the figure indicate the direction of blood flow within the chambers of the heart.

Following surgical intervention, a follow-up echocardiogram demonstrated a significant reduction in LVOT gradients, with a maximum provoked gradient of 38 mmHg. Furthermore, the mitral regurgitation improved substantially from severe to mild. However, the study also revealed increased filling pressures consistent with Grade III diastolic dysfunction, evidenced by an E/e’ ratio of 19. Optimal medical therapy was administered post-surgery and maintained during cardiology follow-up. The established treatment regimen continues to include rivaroxaban for anticoagulation and bisoprolol for rate control. Additionally, an SGLT2 inhibitor was initiated to optimize management for HFpEF. At 3-month follow-up, the patient remained in sinus rhythm, improved to NYHA functional class II, and had no documented readmissions for heart failure. Figure 6 provides a schematic summary of the fixed and dynamic components of LVOT obstruction in this case, together with the corresponding mechanism-based surgical correction.

## 3. Discussion

Hypertrophic cardiomyopathy is a common heart disease with a prevalence estimated at 1:200–1:500, largely based on phenotype detected by imaging, suggesting that 750,000 Americans may be affected by HCM. Symptomatic hypertrophy based on medical claims data has been estimated at 1:3000 adults in the United States [10].

Echocardiography and CMR are established imaging strategies for clinical HCM diagnosis, based on a hypertrophied, non-dilated left ventricle (LV). In patients with hypertrophic cardiomyopathy, absolute left ventricular wall thickness ranges widely from mild (13–15 mm) to massive (>30 mm) [11]. In hospital cohorts, LVOTO is present in approximately 70% of patients with HCM and may occur at rest or with physiological exercise; the remaining third have non-obstructive forms without the capacity to generate outflow gradients. The presence of a peak LVOT gradient ≥30 mmHg is considered indicative of obstruction, with resting or provoked gradients ≥50 mmHg generally considered the threshold for septal reduction therapy (SRT) [12].

Stress echocardiography is an important diagnostic tool in HCM because it allows assessment of the provocability of LVOTO and the generation of dynamic gradients. When exercise echocardiography is not available or feasible, the Valsalva maneuver may help identify LVOT gradients; in selected cases, pharmacologic provocation with amyl nitrite, isoproterenol, or dobutamine (in this order of preference) may be used to unmask latent obstruction [10]. In the present case, exercise stress echocardiography was not feasible because the patient presented with acute decompensated heart failure and cardiogenic shock, making physiologic stress testing inappropriate and unsafe at the time of initial evaluation.

It is important to diagnose, stratify, and adequately investigate LVOTO, as gradients ≥30 mmHg provide prognostic information related to heart failure progression and worsening dyspnea/NYHA functional class. In our patient, the clinical deterioration to cardiogenic shock in the setting of atrial fibrillation with rapid ventricular response underscores how combined LVOTO can become rapidly destabilizing when diastolic filling shortens and dynamic gradients intensify. This presentation reinforces that, beyond symptom burden, timely recognition of the obstructive substrates (fixed and dynamic) is central to preventing recurrent decompensation and guiding definitive therapy.

Subaortic membrane is a rare congenital lesion and represents the second most common type of aortic stenosis after valvular aortic stenosis. It may go unrecognized in early life, with clinical manifestations sometimes developing later due to progressive LVOTO. Subaortic membrane is characterized by a fibrous or fibromuscular structure located immediately beneath the aortic valve [13]. The condition occurs more frequently in males than in females, with a reported male-to-female ratio of approximately 2:1. When subaortic membrane coexists with HCM, a key diagnostic challenge is disentangling fixed subvalvular obstruction from dynamic obstruction driven by SAM, drag forces, and altered mitral–septal geometry. Echocardiography may demonstrate two discrete levels of obstruction, and a “double-envelope” Doppler pattern has been described as a practical clue to concomitant fixed and dynamic components [6]. Importantly, subaortic membrane can be clinically underappreciated and, if overlooked, may lead to persistent gradients despite therapies aimed at the dynamic component alone [5].

Three morphological subtypes have been described: (1) discrete subaortic stenosis, typically presenting as a thin membranous structure and accounting for 75–85% of cases; (2) a thick fibromuscular ridge; and (3) a tunnel-like or tubular form of obstruction [5,6].

Subaortic membrane is an uncommon cause of LVOTO, and its hallmark is fixed (subvalvular) obstruction, in contrast to the dynamic obstruction characteristic of HCM (typically mediated by SAM, drag forces, and abnormal mitral–septal interaction). Transthoracic echocardiography is usually the first diagnostic step, but it may miss a subaortic membrane; therefore, multimodality imaging is critical for identifying the etiology of LVOTO. Transesophageal echocardiography plays a crucial role when the mechanism of LVOTO is not clearly identified by transthoracic imaging, particularly when alternative or coexisting causes of obstruction are suspected [2].

Cardiac catheterization represents an alternative modality for clarifying the mechanism and severity of subaortic obstruction, providing direct hemodynamic assessment, including measurement of pressure gradients and cardiac output, particularly in cases in which echocardiographic evaluation is inconclusive [2].

We promote the use of multimodality imaging, including CMR and cardiac computed tomography when available. CMR provides high spatial and temporal resolution; in HCM it can precisely quantify septal thickness, identify hypertrophy in segments that echocardiography may miss (e.g., apex or anterolateral free wall), determine scar burden (fibrosis), and support septal reduction planning. Cardiac computed tomography offers high anatomic resolution and can clarify LVOT anatomy; it is also important before surgery to exclude coronary disease and assess the need for coronary intervention [2,3]. A further reason to emphasize multimodality imaging is that serial obstructive lesions can complicate Doppler-based quantification, because the modeling assumptions of the modified Bernoulli equation may be less reliable when stenoses occur in series. This was particularly relevant in our case, because the retrospective echocardiographic archive did not preserve separate serial Doppler envelopes or post-extrasystolic measurements, precluding precise quantification of the relative hemodynamic contribution of each obstructive level. Although this concept is classically discussed for concomitant LVOTO and aortic stenosis, the same hemodynamic principle supports a careful stepwise evaluation when fixed and dynamic obstructions coexist within the LVOT [9]. In our case, the combination of TTE/TEE with CT and CMR allowed comprehensive anatomical definition (membrane morphology and septal hypertrophy), exclusion of coronary disease, and tissue characterization (LGE 13%), strengthening both diagnostic certainty and surgical planning [7]. The 13% late gadolinium enhancement burden also raises broader considerations regarding sudden cardiac death risk stratification in HCM. However, because this was a retrospective case report with incomplete longitudinal arrhythmic data, we did not attempt to infer ICD candidacy from this case alone.

The correction of subaortic obstruction includes simple membrane removal, extensive ring resection with or without myectomy, or a Konno procedure [14]. In parallel, the therapeutic landscape of obstructive hypertrophic cardiomyopathy has evolved with the emergence of targeted pharmacological therapies. In particular, Mavacamten, a first in class cardiac myosin inhibitor, has demonstrated significant reductions in LVOT gradients and improvement in functional capacity and symptoms in patients with obstructive HCM, as shown in randomized clinical trials [15,16].

In the EXPLORER-HCM trial, mavacamten improved symptoms, NYHA class, and quality of life in patients with obstructive HCM [15]. In the VALOR-HCM trial, it reduced LVOT gradients and the need for septal reduction therapy in eligible patients [16]. Although less invasive or combined strategies may be considered in selected cases, the present patient had clinically significant fixed and dynamic LVOT obstruction, severe SAM-related mitral regurgitation, and hemodynamic instability. In addition, long-term access to targeted therapy may be limited in some settings. Accordingly, the Heart Team selected a mechanism-based surgical strategy consisting of subaortic membrane resection for the fixed component, septal myectomy for the dynamic HCM-related component, and adjunctive mitral valve plication for severe SAM-related MR in the setting of annular dilatation. This integrated approach is mechanistically important because septal reduction alone would not relieve membrane-related fixed obstruction, whereas membrane resection alone would not fully treat the dynamic SAM-mediated gradient in obstructive HCM [5,6]. Unlike several published cases in which conservative management was chosen or follow-up was limited, we document objective early postoperative improvement in LVOT gradients and SAM-related MR severity, suggesting that comprehensive surgical correction may be appropriate when both obstructive substrates are clinically significant [6,8].

The coexistence of these two distinct entities is extremely rare, and this association represents a significant clinical challenge that requires a high index of suspicion. Even in experienced centers, establishing an accurate diagnosis may be difficult; however, correct diagnostic differentiation is essential for appropriate therapeutic decision-making and selection of optimal intervention.

This report has important limitations. First, the retrospective echocardiographic archive did not allow complete Doppler separation of the relative hemodynamic contribution of each obstructive level. Second, detailed intraoperative morphological descriptors and immediate post-bypass hemodynamic measurements were not fully available in the archived record. Third, follow-up was limited and partly based on remote clinical contact after the patient relocated.

## 4. Conclusions

Hypertrophic cardiomyopathy complicated by both dynamic LVOTO and coexisting fixed subvalvular obstruction due to a subaortic membrane is exceedingly rare. Accurate diagnosis requires a high index of suspicion, and the use of multimodality imaging plays a pivotal role in defining the underlying mechanisms of obstruction in our patient, directly informing the timing and nature of the surgical intervention. We suggest that in cases where the etiology of LVOTO remains uncertain, multimodality imaging should be considered to guide therapeutic decision-making.

Although the association between HCM and severe mitral regurgitation secondary to SAM is well established, careful characterization of the regurgitation mechanism remains essential to optimize therapeutic planning. In the present case, a Heart Team-guided surgical approach consisting of septal myectomy, subaortic membrane resection, and adjunctive mitral valve plication was associated with a favorable early postoperative outcome. This case also highlights the clinical importance of recognizing a coexisting fixed subvalvular lesion, since treatment directed only at the dynamic component may lead to incomplete relief of LVOT obstruction and persistent residual gradients. Given the limited follow-up duration and the complexity of the underlying pathology, longer-term surveillance will be necessary to assess the durability of surgical repair and the risk of recurrence.

## Figures and Tables

**Table 1:** Baseline laboratory profile (clinical and hematologic parameters) of the patient.

Parameter	Result	Reference Range
White blood cell count (×10^3^/µL)	7.21	3.4–9.7
Neutrophils (×10^3^/µL)	5.82	2.2–4.8
Lymphocytes (×10^3^/µL)	1.30	1.1–3.2
Hemoglobin (g/dL)	13.1	12.0–16.0
Platelets (/µL)	274	130,000–400,000
Troponin T (ng/mL)	0.03	0–0.01
NT-proBNP (pg/mL)	8334	<125
Creatinine (mg/dL)	1.1	0.74–1.35
CA-125 (U/mL)	99.7	0–35

## Data Availability

The original contributions presented in this study are included in the article. Further inquiries can be directed to the corresponding author.
